# Caregiver preferences for physically harsh discipline of children in rural Uganda

**DOI:** 10.1007/s10896-023-00536-4

**Published:** 2023-04-01

**Authors:** Emily N. Satinsky, Bernard Kakuhikire, Charles Baguma, Christine E. Cooper-Vince, Justin D. Rasmussen, Scholastic Ashaba, Jessica M. Perkins, Phionah Ahereza, Patience Ayebare, Andrew W. Kim, Eve S. Puffer, Alexander C. Tsai

**Affiliations:** 1 Center for Global Health, Massachusetts General Hospital, Boston, MA, USA; 2 Department of Psychology, University of Southern California, Los Angeles, CA, USA; 3 Mbarara University of Science and Technology, Mbarara, Uganda; 4 Départment de Psychiatrie, Universitié de Genève, Geneva, Switzerland; 5 Department of Psychology, Duke University, Durham, NC, USA; 6 Peabody College, Vanderbilt University, Nashville, TN, USA; 7 Department of Anthropology, University of California, Berkeley, CA, USA; 8 Harvard Medical School, Boston, MA, USA

**Keywords:** Corporal punishment, Discrete choice task, Economic insecurity, Physically harsh discipline, Uganda

## Abstract

**Purpose:**

Physically harsh discipline is associated with poor developmental outcomes among children. These practices are more prevalent in areas experiencing poverty and resource scarcity, including in low- and middle-income countries. Designed to limit social desirability bias, this cross-sectional study in rural Uganda estimated caregiver preferences for physically harsh discipline; differences by caregiver sex, child sex, and setting; and associations with indicators of household economic stress and insecurity.

**Method:**

Three-hundred-fifty adult caregivers were shown six hypothetical pictographic scenarios depicting children whining, spilling a drink, and kicking a caregiver. Girls and boys were depicted engaging in each of the three behaviors. Approximately half of the participants were shown scenes from a market setting and half were shown scenes from a household setting. For each scenario, caregivers reported the discipline strategy they would use (time out, beating, discussing, yelling, ignoring, slapping).

**Results:**

Two thirds of the participants selected a physically harsh discipline strategy (beating, slapping) at least once. Women selected more physically harsh discipline strategies than men (b = 0.40; 95% confidence interval [CI], 0.26 to 0.54). Participants shown scenes from the market selected fewer physically harsh discipline strategies than participants shown scenes from the household (b = −0.51; 95% CI, −0.69 to −0.33). Finally, caregivers selected more physically harsh discipline strategies in response to boys than girls. Indicators of economic insecurity were inconsistently associated with preferences for physically harsh discipline.

**Conclusions:**

The high prevalence of physically harsh discipline preferences warrant interventions aimed at reframing caregivers’ approaches to discipline.

## Introduction

Violence against children is prevalent globally, with over half of all children between the ages of 2 and 17 experiencing violence in the past year (Hillis et al. 2016). Comprised of six main types of experiences, violence against children may include sexual violence (e.g., rape, sexual trafficking, sexual harassment, online exploitation); intimate partner violence (including among girls in child and early/forced marriages); youth violence (i.e., that which occurs between children and young adults, with or without weapons); bullying involving repeated harm; emotional or psychological violence (i.e., that which ridicules, intimidates, and rejects children through non-physical hostility); and child maltreatment (i.e., that which involves any type of neglect, abuse, or violent punishment, usually within schools or the household) (World Health Organization, 2020). Across the range of experiences, violence against children is associated with detrimental outcomes. An extensive literature describes pathways from childhood sexual abuse, physical abuse, and other forms of maltreatment to severe psychopathology during childhood, adolescence, and adulthood (Albott et al. 2018; Ashaba et al. 2021, 2022; Cluver et al. 2015; Dube et al. 2001; Hailes et al. 2019; Meinck et al. 2015; Negriff 2020; Satinsky et al. 2021).

Under the umbrella of child maltreatment, physical punishment comprises disciplinary practices that inflict any degree of physical pain or discomfort on the child. It has been described as a form of violence that degrades children and compromises their right to protection (United Nations Committee on the Rights of the Child 2006). Even if it does not rise to the level of malevolent physical abuse, physically harsh discipline is associated with negative developmental, social, and psychological outcomes (Bender et al. 2007; Cuartas 2021; Durrant and Ensom 2012; Gershoff and Grogan-Kaylor 2016; Heilmann et al. 2021). Children exposed to physically harsh discipline practices exhibit lower educational outcomes (Smith and Brooks-Gunn 1997) and greater peer victimization (Ssenyonga et al. 2019), physical aggression (Kingsbury et al. 2020), behavioral problems (Mackenbach et al. 2014; Mulvaney and Mebert 2007; Parent et al. 2011), and depression (Hecker et al. 2016). In response to physical punishment, children’s externalizing and internalizing behaviors worsen over time (Heilmann et al. 2021). Functioning in a cyclical nature, these child behaviors may thereby increase caregivers’ use of physically harsh discipline. Furthermore, individuals who are exposed to physical punishment during childhood are more likely to perpetrate similar practices toward their own children (Fulu et al. 2017; Milner et al. 1990; Simons et al. 1991). The consequences of physical punishment are not confined to childhood. Prior studies have found associations between physical punishment during childhood and poor economic (Henry et al. 2018), behavioral (Afifi et al. 2019), and health outcomes (Felitti et al. 1998; Hughes et al. 2017; Jewkes et al. 2010; Kessler et al. 2010) during adulthood.

Some research shows a higher prevalence of physically harsh discipline in rural settings (Alyahri and Goodman 2008), and evidence from diverse contexts has indicated robust associations between poverty and exposure to physically harsh discipline in childhood (Dietz 2000; Drake and Pandey 1996; Ricketts and Anderson 2009). Moreover, the prevalence of physical punishment among children is greater in low- and middle-income countries than in high-income countries (Hillis et al. 2016). Research suggests that other measures of household insecurity, including food and water insecurity, are associated with both caregiver stress and physically harsh discipline (Helton et al. 2018; Huang et al. 2016).

People living in rural areas of Uganda face pervasive poverty and resource insecurity (Mushavi et al. 2020; Perkins et al. 2018; Tsai et al. 2011; World Bank 2016). Child poverty is particularly prevalent in these areas, with slower progress in household and child-specific indicators when compared to progress in more urban areas (Wasswa 2015). In 1997, Uganda banned corporal punishment in schools (End Violence Against Children, 2022); in 2016, Sect. 106A of The Children (Amendment) Act further prohibited corporal punishment in any institution of learning, with offenders liable to imprisonment (Republic of Uganda, 2016). Physical punishment, however, remains normative in both schools and households (UNICEF 2018). In a national survey from Uganda conducted in 2015, nearly half of children reported experiencing physically harsh discipline at the hand of a parent, caregiver, or other adult relative (UNICEF 2018).

Despite evidence indicating a high prevalence of physical punishment in Uganda, little is known about the extent to which physically harsh discipline practices vary by contexts outside of school settings and how much household economic stress and insecurity drive preferences for these practices. Thus, we conducted a cross-sectional survey to better understand patterns of preferences for physically harsh discipline of young children in rural Uganda, as well as preferences by caregiver sex, child sex, and setting. We also aimed to estimate the associations between indicators of economic security and physically harsh discipline preferences.

## Methods

### Study setting, population, and procedure

This study took place in Nyakabare Parish, Rwampara District, a rural region in southwestern Uganda. This region was selected through an iterative process. Field site visits to parishes within Mbarara District and informal conversations with local leaders and prominent village residents guided our team’s selection. Nyakabare Parish was favored over other regions due to the limited presence of nongovernmental organizations in the area, the general similarity of the parish compared with other rural parishes in the region, and local leaders’ support for engaging residents in a household survey. The parish is comprised of 8 villages and is located about a five-hour drive from the capital, Kampala. Most parish residents work in subsistence farming, animal husbandry, and/or small-scale enterprise, and many report insecure access to food and water (Mushavi et al. 2020; Perkins et al. 2018; Tsai et al. 2011).

The present study took place within the context of an ongoing population cohort (Takada et al. 2019). Rather than interviewing all caregivers in the parish, and to minimize participant burden, we targeted a convenience sample of 350 study participants of both sexes. Given the study’s focus on understanding the hypothetical circumstances under which a caregiver would express a preference for harsh disciplinary strategies, the subsample of study participants was not randomly selected from the population. In this sub-study, adults 18 years or older were eligible for participation if they or their partner had a biological child 4–12 years of age who primarily stayed in their household. We were interested in this sub-population specifically, rather than adults in general, because of our specific interest in caregivers’ preferences for physically harsh discipline of young children. Individuals unable to adequately communicate with the research team due to cognitive impairment, behavioral problems, deafness, or mutism were excluded.

The study was conducted in 2019–2020. Prior to under-taking study procedures, we held a number of community engagement meetings to introduce the study to parish residents and solicit feedback (Kakuhikire et al. 2021). For this study, a team of research assistants visited the homes of all eligible adults. Research assistants requested study participation and participants provided written informed consent prior to beginning the interview. Those who were unable to read or write were permitted to indicate consent with a thumbprint mark. To administer the survey, the research assistant and participant moved to a private area in or near the participant’s home. Interviews were conducted in Runyankore, the local language, and data were collected with a survey collection software program that could be used remotely with limited access to the internet. Following initial development in English, research assistants translated the measures into Runyankore. Subsequently, other research assistants on the team back-translated the measures into English to confirm fidelity of the translations. This process proceeded iteratively and involved consultation with key informants and pilot testing.

### Measures

A survey instrument was adapted from Green et al. (2017) to measure participants’ caregiving discipline preferences. The original instrument was developed for use in a discrete choice experiment conducted in Liberia (Green et al. 2018), with the aim of limiting social desirability bias in participant responses and minimizing potential underestimates of prevalence. Some images from the original instrument were redrawn in accordance with research assistant feedback to improve interpretability. Participants were first presented with pictographic images of different scenarios to confirm understanding of key concepts. The first set of three images depicted child behaviors: 1) whining, 2) spilling a drink, and 3) kicking a caregiver. The second set of images depicted six possible disciplinary actions taken in response to these behaviors: 1) putting the child in “time out”, 2) beating the child, 3) discussing the behavior with the child, 4) yelling at the child, 5) ignoring the child, and 6) slapping the child. After viewing each of the nine images (3 child behaviors, 6 discipline strategies), the participant was asked to describe what was happening in the image. If the participant expressed uncertainty about what was happening, the research assistant explained the image until the participant confirmed understanding. If the research assistant was unable to confirm the participant’s understanding of any of the nine images, that participant was excluded from the remainder of the study procedures described below.

**Fig. 1:**
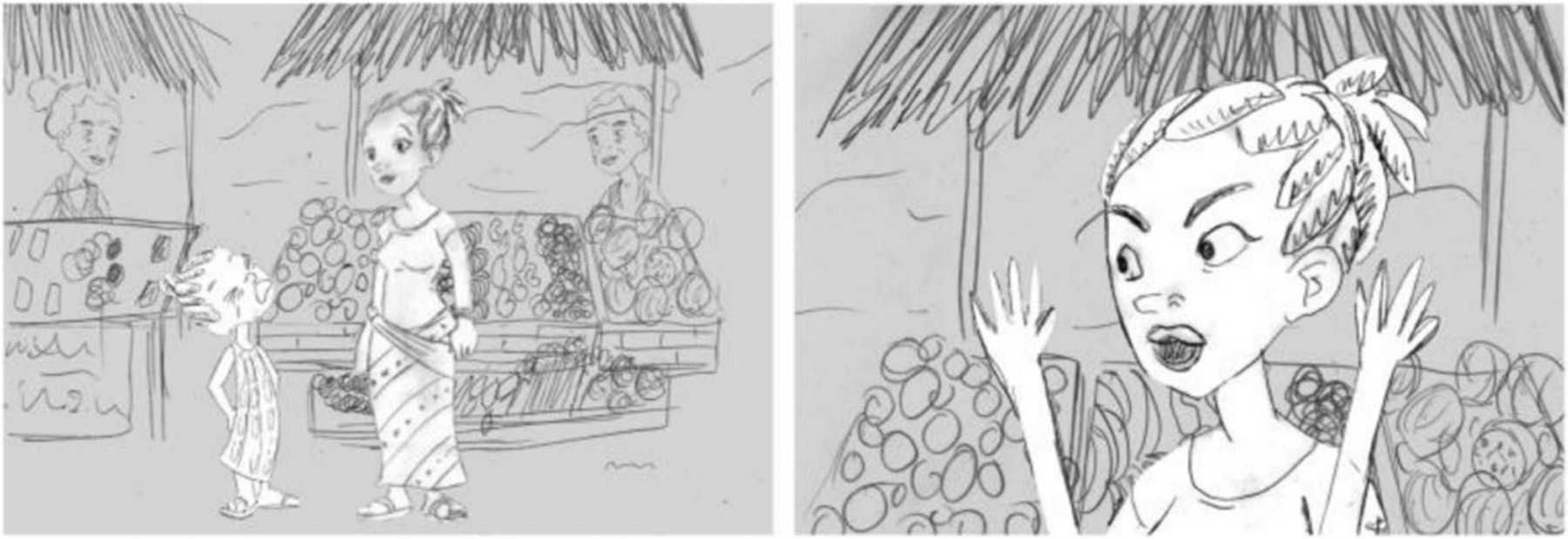
A girl is whining to a female caregiver in a market setting

The research assistant presented each participant with six pictographic comic strips, each with an image of a child engaging in a particular behavior (Fig. 1). Three of the six comic strips depicted a girl engaging in one of the prespecified behaviors (whining, spilling a drink, kicking a caregiver), and the other three depicted a boy engaging in the same behaviors. Although the age of the children in the comic strips was unspecified, the images were intended to represent children between the ages of 4 and 12. The order of the scenarios was mixed (but not randomly assigned) such that some participants were shown a girl engaging in the behavior first, while other participants were shown a boy first.

**Fig. 2:**
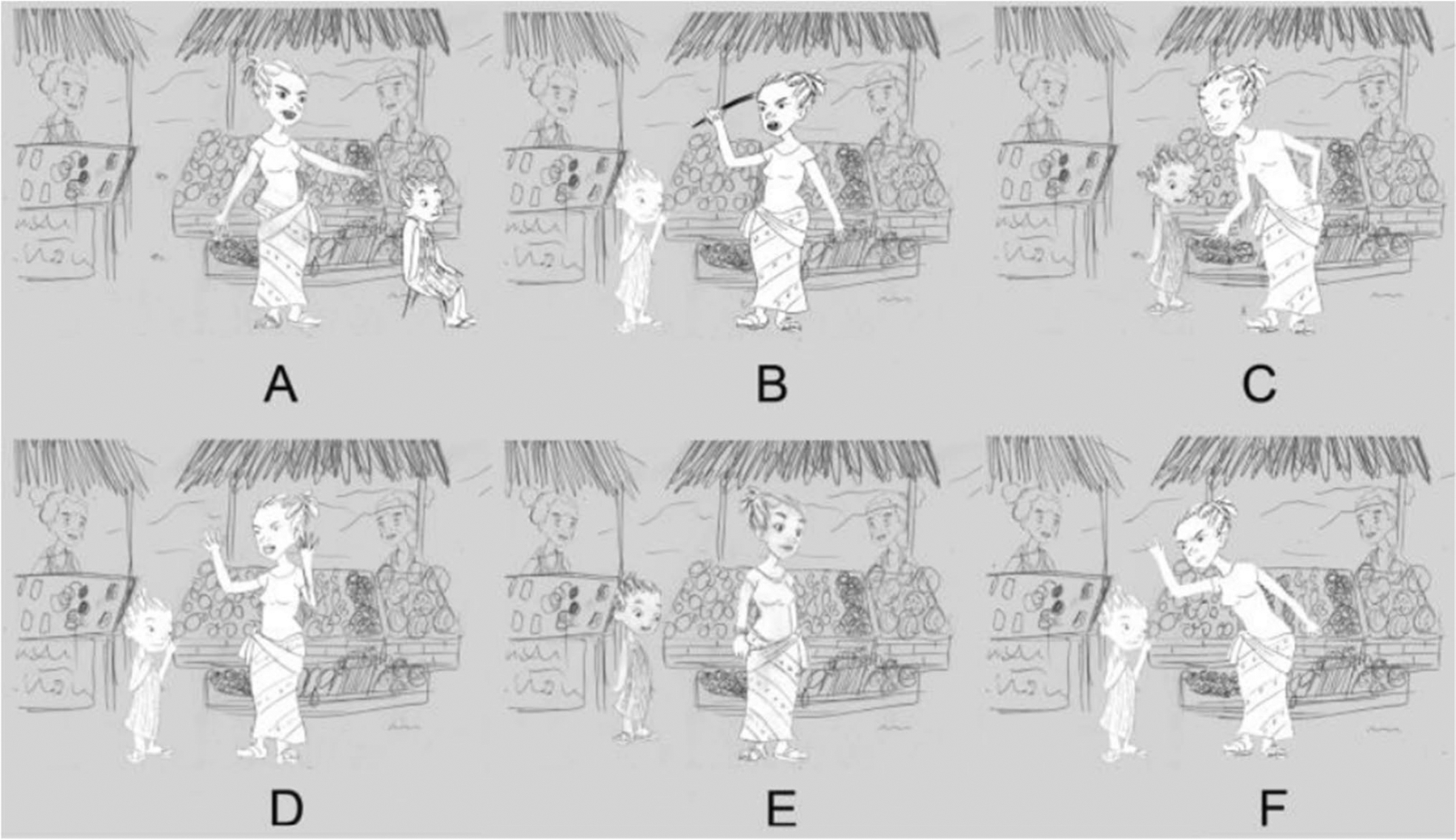
Possible response options: (**A**) time out, (**B**) beating, (**C**) discussing, (**D**) yelling, (**E**) ignoring, (**F**) slapping

The research assistant then showed the participant images of a caregiver responding to the child using one of the six discipline strategies (time out, beating, discussing, yelling, ignoring, slapping; Fig. 2). The sex of the caregiver depicted in the comic strip was concordant with the study participant’s sex, i.e., women were shown a comic strip depicting a woman caregiver, while men were shown a comic strip depicting a man caregiver. The research assistant asked the participant to select the image that most closely resembled how they would respond in the depicted scenario (i.e., to the child’s behavior; Fig. 2).

To ensure that study participants’ choices of discipline strategies were not driven by a single setting, we presented participants with comic strips depicting scenes from either a household or an open-air, local public market. Allocation to the vignettes was not randomly assigned. Instead, we administered the version of the vignettes set in the household setting until approximately half of the targeted sample size had been interviewed, and we then administered the version of the vignettes set in the market setting to the remainder of the sample. While the household is a private setting where families spend substantial time, the market represents a common public space. Ugandan families in both rural and urban regions visit markets to trade, buy, and sell goods and interact with other village members.

For each scenario, participants were categorized as endorsing physically harsh discipline if they selected ‘beating’ or ‘slapping.’ The primary outcome of interest (i.e., preferences for physically harsh discipline practices) was based on summing responses across the six scenarios to create a variable indicating the total number of scenarios (out of 6) in which the participant selected a physically harsh discipline strategy. Two additional variables were calculated, representing the total number of scenarios in which the participant selected a physically harsh discipline strategy in response to a boy (out of 3) and in response to a girl (out of 3).

The primary explanatory variables of interest were four measures of economic security. Food insecurity was assessed with the 9-item Household Food Insecurity Access Scale (Perkins et al. 2018; Swindale and Bilinsky 2006). Water insecurity was assessed using the 8-item Household Water Insecurity Access Scale (Mushavi et al. 2020; Tsai et al. 2016). Participants were also asked to report whether or not they owned different assets in their household (e.g., goats, chickens, radio, bike, car, flush toilet or ventilated improved pit latrine, etc.). Principal component analysis was applied to categorize participants into household asset wealth quintiles (Filmer and Pritchett 2001; Smith et al. 2020). Finally, participants reported their self-perceived relative wealth, i.e., how wealthy they perceived themselves to be in relation to other members of the parish (least poor, better off, average, worse off, or poorest) (Smith et al. 2019).

### Ethical approval

All research assistants received multi-day trainings on how to administer surveys for gathering sensitive information, including explicit instructions on how to temporarily halt the survey without arousing suspicion if another person came within earshot. The research assistants also received two additional training courses, one from The AIDS Support Organization on how to handle study participant reports of interpersonal violence (namely, how to receive the information in a sensitive manner and how to provide appropriate referrals to domestic protection resources) and one from a Ugandan counseling psychologist about how to manage sensitive disclosures by study participants. Feedback on the study design was solicited from a community advisory board comprised of eight community leaders, including four women and the district community development officer. We received ethical approval from the Mbarara University of Science and Technology Research and Ethics Committee and the Partners Human Research Committee. We also obtained clearance from the Uganda National Council for Science and Technology.

### Data analysis

We tabulated preferences for physically harsh discipline across all three child behaviors, stratified by caregiver sex, child sex, and setting, separately. We then conducted chi-squared tests to compare discipline preferences (i.e., reported any physically harsh discipline strategies vs. none) by caregiver sex and setting. These analyses were conducted separately for boys and girls. Since the total harsh discipline strategy count variable was non-normally distributed and over-dispersed, we fit negative binomial regression models to estimate the associations between preferences for physically harsh discipline (total count) and caregiver sex and setting.

We also fit a series of negative binomial regression models to estimate associations between indicators of economic security and preferences for physically harsh discipline (total count). We fitted separate models for each of the four economic variables: food insecurity, water insecurity, household asset wealth quintile, and self-perceived relative wealth. All models were fitted with cluster-correlated robust estimates of variance to account for potential within-village clustering of the outcome and adjusted for age, caregiver sex, primary school completion, marital status, and setting (i.e., household vs. market). In sensitivity analyses, we stratified these analyses by the sex of the child depicted in the scenario.

As a robustness check to assess the extent to which the convenience sampling may have yielded biased estimates compared to a whole-population sample or a random sample of the population, we used inverse probability of treatment weights (IPTWs) to recover estimates that would be representative of all Nyakabare Parish residents with children under the age of 18 living in their household. First we fitted a logistic regression model, where inclusion in the subsample of 350 caregivers was specified as the dependent variable, and we included variables that were potentially correlated with participation: age, sex, education level, village of residence, household asset wealth, self-reported overall health, index of social participation, water insecurity, and food insecurity. We then used this regression model to calculate stabilized IPTWs (Cole and Hernán 2008; Hernán et al. 2000; Robins et al. 2000). We incorporated these IPTWs into the regression models specifying preferences for harsh discipline as the dependent variable. Analyses were conducted in Stata MP (version 16, StataCorp LLC, College Station, Tex.).

## Results

Three hundred and fifty-three caregivers were recruited to participate in this study. Two individuals did not understand the ‘time out’ discipline strategy and one did not understand the ‘yelling’ discipline strategy, even after discussing the image with the research assistant. These three participants were therefore excluded from further study procedures and from the analysis. In all, 350 caregivers completed the study (Table 1). Of these, 198 (57%) were men. Most participants had completed primary school (n = 221, 63%) and were married (n = 321, 92%). The caregivers in the sample were distributed among 270 households: 191 households provided 1 participant, 78 households provided 2 participants, and 1 household provided 3 participants. Most participants reported mild to severe food insecurity (n = 212, 61%) and more than a third reported mild to severe water insecurity (n = 126, 36%). While just over half of the sample perceived that they were at least equally as well off as other members of the parish (n = 176, 50%), about a quarter believed that they were worse off (n = 90, 26%).

There were no statistically significant differences between participants shown scenes from the household vs. market setting across the following demographic characteristics: age, education level, marital status, household water insecurity, household asset wealth quintile, and self-perceived relative wealth. However, participants shown scenes from the household setting were more likely to report household food insecurity compared with participants shown scenes from the market setting (69% vs. 53%, χ^2^ = 18.93, *p* < 0.001).

Across all six scenarios, 77% of women and 59% of men selected a physically harsh discipline strategy at least once. Participants selected a physically harsh discipline strategy 2.21 times on average (standard deviation [SD] = 2.02, median = 2, interquartile range = 0–4) out of a possible six. Generally, caregivers were more likely to select a physically harsh discipline strategy in the comic strips depicting a boy (Table 2; Supplementary File 1). Additionally, caregivers were least likely to select a physically harsh discipline strategy if the child was depicted whining and more likely to select a physically harsh discipline strategy if the child was depicted kicking the caregiver.

Overall, 179 participants (51%) were presented with scenes from a market setting, and 171 participants (49%) were presented with scenes from a household setting. Women were no more likely than men to select a physically harsh discipline strategy in market settings. However, women were more likely than men to select at least one physically harsh discipline strategy in household settings. Compared to only 59% of men (n = 43), 83% of women (n = 81) selected at least one physically harsh discipline strategy for use with girls in the household (χ^2^ = 11.8, *p* = 0.001). Similarly, 84% of women (n = 82) and 66% of men (n = 48) selected at least one physically harsh discipline strategy for use with boys in the household (χ^2^ = 7.37, *p* = 0.007).

In negative binomial regression models with indicators for caregiver sex, women were more likely than men to select physically harsh discipline strategies overall (b = 0.40, 95% confidence interval [CI], 0.26 to 0.54, *p* < 0.001; with inverse probability [IP] weights: b = 0.42, 95% CI, 0.22 to 0.62, p < 0.001). When compared with the baseline standard deviation, the predicted mean difference between women and men was (2.71–1.82)/2.02 = 0.44 SD units, or 40% relative to the baseline overall mean. Participants who were shown scenes from the market setting selected fewer physically harsh discipline strategies than participants who were shown scenes from the household setting (b = −0.51, 95% CI, −0.69 to −0.33, *p* < 0.001; with IP weights: b = −0.48, 95% CI, −0.79 to −0.16, p = 0.003). When compared with the baseline standard deviation, the predicted mean difference between household and markets settings was (2.77–1.66)/2.02 = 0.55 SD units, or 50% relative to the baseline overall mean.

In the multivariable negative binomial regression models, none of the indicators of economic security were reliably associated with preferences for physically harsh discipline (Table 3; Supplementary File 2). The signs of the estimated coefficients were not consistent across indicators: food insecurity was positively associated with preferences for physically harsh discipline (*p*-values ranging from 0.06 to 0.28), while water insecurity had null associations (regression coefficients ranging from −0.07 to 0.02 and *p*-values ranging from 0.72 to 0.91); and similarly, being in the poorest quintile as measured by asset wealth was positively associated with preferences for physically harsh discipline (*p* = 0.07) but being in the poorer categories of self-perceived wealth was negatively associated (*p*-values ranging from 0.03 to 0.44). Similarly, indicators of economic security were inconsistently associated with the cumulative number of physically harsh discipline preferences when the analyses were stratified by child sex (Supplementary File 3).

## Discussion

In this study, over two-thirds of caregivers selected a physically harsh discipline strategy at least once when presented with pictographic images of hypothetical child behaviors in a household or market setting. While the prevalence of preferences for physically harsh discipline was high across contexts, these preferences were more common among women caregivers compared to men caregivers and were more common in response to behaviors in the household setting compared to the market setting. These estimates were small to moderate in magnitude. Generally, caregivers selected harsh discipline strategies in scenes depicting boys more frequently than in scenes depicting girls. Finally, within this rural setting with insecure access to food and water (Mushavi et al. 2020; Perkins et al. 2018; Tsai et al. 2011), physically harsh discipline preferences were not reliably associated with indicators of economic security.

These findings reflect research from other settings across eastern and southern Africa that show a high prevalence of physically harsh discipline, both in the school and household (Devries et al. 2014; Lansford et al. 2010; O’Neil et al. 2009). Preferences for physically harsh discipline in the present study were consistent with national survey data from Uganda (Uganda Burea of Statistics 2018; UNICEF 2018). For example, in the Uganda 2016 Demographic and Health Survey, 68.8% of respondents from the southwestern region, which includes Mbarara District, endorsed the belief that physical punishment is needed to raise a child properly (Uganda Burea of Statistics 2018). As the present study was designed specifically to limit response bias (Green et al. 2017, 2018), our data confirm a high prevalence of preferences for physical punishment in this setting. The use of hypothetical, pictographic scenarios allowed caregivers to describe their preferences while not having to report actual practices. Although physically harsh discipline remains normative in this setting (Lokot et al. 2020; Ssenyonga et al. 2019), caregivers may still be reluctant to report their true discipline practices. To the extent that social desirability bias remains an important predictor of reporting, other methods (e.g., list experiments; Lépine et al. 2020; Moseson et al. 2015) may elicit even more accurate estimates of such preferences.

Our findings build on prior research by demonstrating differences in preferences for physically harsh discipline by caregiver sex, setting, and child sex. A global study across nine countries found that mothers were more likely than fathers to use corporal punishment. Similarly, boys were more likely than girls to be disciplined through physical means (Lansford et al. 2010). In Uganda, women are more likely to assume responsibility for caregiving and domestic work, while men are more likely to work outside of the home (Gabola et al. 2018). As such, women caregivers spend more time with the children and take on the role of disciplinarian. During the limited time they spend with their children, and to compensate for their long absences, men might be less likely to use physically harsh discipline. Gendered norms and idealized masculinity in this setting may further decrease the likelihood that men engage in caregiving activities, including setting boundaries and disciplining children.

The more limited prevalence of physically harsh discipline preferences in the market setting versus the household setting may reflect a preference for delaying physically harsh discipline until the caregiver and child are in a secluded location. Caregivers may be less likely to subject their child to physically harsh discipline in public settings due to stigma associated with such behavior, particularly in light of the ban on corporal punishment in schools (Republic of Uganda 2016). Even though the use of physically harsh discipline is widely practiced and culturally accepted in Uganda (Lokot et al. 2020; Ssenyonga et al. 2019), caregivers may not want others to witness them using these practices. Descriptive norms (i.e., what most people think, feel, or do) might not reflect injunctive norms (i.e., what most people approve of) in this context. Nonetheless, prior studies have shown a strong association between stated preferences for harsh discipline and engagement in harsh disciplinary behaviors (Johnson et al. 2022).

Unlike past research, we did not find that men or women living in conditions of economic insecurity (i.e., potentially fostering more parenting stress) were more likely to report preferences for physically harsh discipline (Johnson et al. 2022). This finding may reflect cultural norms around the use of physically harsh discipline that hold across socioeconomic strata. Additionally, since this study was conducted in a rural setting characterized by considerable food and water insecurity (Mushavi et al. 2020; Perkins et al. 2018; Tsai et al. 2011), associations between economic insecurity and harsh discipline may extend throughout the entire study setting. In a qualitative study of 12 mothers living in poverty in Kampala, Uganda, for example, women described using corporal punishment with their children to ensure good behavior, manage scarce resources, and protect their children from health risks (Boydell et al. 2017). Future research may consider sampling caregivers from multiple regions of the country to assess relationships between wealth and discipline in settings with greater economic variability.

The high prevalence of preferences for physical punishment in this setting is cause for concern. Although there is a spectrum of physically harsh discipline strategies, ranging from malevolent behavior intentionally used to cause physical harm to the occasional spanking (World Health Organization, 2020), the use of strategies at all points along this continuum are associated with poor developmental outcomes (Cuartas 2021; Gershoff and Grogan-Kaylor 2016; Hecker et al. 2016; Kingsbury et al. 2020; Mackenbach et al. 2014). While some research indicates that normative perceptions of physically harsh discipline partially moderate the relationship between physical punishment and detrimental outcomes (Gershoff et al. 2010; Lansford et al. 2005), other research finds that physically harsh discipline negatively affects development, even in cultures where these practices are perceived as normative (Pace et al. 2019; Yildrim et al. 2020). Thus, our findings support the need for intervention strategies that encourage alternative, non-physically harsh discipline (Giusto et al. 2020; Puffer et al. 2020).

Parenting resources available to caregivers in Uganda are extremely scarce, particularly among those living in rural settings. Developmentally appropriate interventions that emphasize nurturing care are needed (Black et al. 2017, 2021). These types of interventions have the potential to change norms and attitudes around discipline (Ashburn et al. 2017; Kyegombe et al. 2017; Lokot et al. 2020), reduce the use of physically harsh discipline, and minimize negative developmental outcomes among children. Parenting interventions, potentially led by peers (Giusto et al. 2021a), that promote non-physical discipline strategies and encourage caregivers to discuss boundaries and consequences may help parents better support and empower their children. Although physically harsh discipline preferences were more prevalent among women, interventions should include fathers or other male caregivers (Ashburn et al. 2017; Giusto et al. 2021b; Giusto and Puffer 2018; Lundahl et al. 2007). Several such interventions have been implemented in eastern, western, and southern Africa and have led to reductions in physically harsh discipline practices, improvements in positive parenting, and improvements in child, adolescent, and caregiver outcomes (Ashburn et al. 2017; Betancourt et al. 2014; Cluver et al. 2016, 2018; Lachman et al. 2017; Puffer et al. 2015; Siu et al. 2017; Ward et al. 2019).

### Limitations

First, our findings may not generalize outside of this study setting. However, as our estimates of the overall prevalence of physically harsh discipline preferences mirror the findings on the prevalence of physical abuse and corporal punishment across eastern Africa, it is likely that our findings are reflective of prevalence rates in other rural settings in the region. Second, the limited sample size prevented us from disaggregating analyses by type or severity of discipline strategy (i.e., beating vs. slapping), or considering associations with harsh, yet non-physical punishment (i.e., yelling). Further, the sample size prevented us from estimating potentially meaningful effect sizes. For example, in the analysis of preferences for harsh discipline by household asset wealth quintile, at the means of the covariates the predicted number of scenarios in which a study participant selected a harsh discipline strategy was 2.45 among study participants in the poorest wealth quintile and 2.0 among study participants in the least poor wealth quintile. This represents a 0.45/2.02 = 0.22 SD units difference, or a “small” effect size. While this analysis, for example, could rule out statistically significant larger effect sizes, we could not exclude the possibility of smaller effect sizes. Third, although we estimated associations between discipline preferences and economic security, we did not collect data on child outcomes, including development, school performance, mental health, or physical health. Future research from this setting may include child measures to link with data on caregiver discipline. Fourth, exposure to the vignettes in the household setting vs. vignettes in the market setting was not randomly assigned. Study participants in the two groups were largely similar but did differ in the proportions reporting food insecurity. In the multivariable regression models, which adjusted for household vs. market setting, food insecurity did not have a statistically significant association with preferences for physically harsh discipline, but the *p*-values for the food insecurity categories were closer to the alpha 0.05 threshold (ranging from 0.06 to 0.28). It is possible that there were unobserved differences in socioeconomic status (not captured by differences in food insecurity, water insecurity, household asset wealth, and self-perceived relative wealth) differential by setting that could have contributed to the observed differences in preferences for physically harsh discipline. Fifth, our use of a convenience sampling strategy to identify participants for this sub-study may have introduced bias. For example, it is possible that unobserved characteristics associated with the propensity to participate in the sub-study may have been correlated with both economic insecurity and with preferences for physically harsh discipline in such a way as to bias the estimated associations either toward or away from the null. However, it is reassuring that IP weighted estimates – representative of all parish residents with a child under the age of 18 living in the household – did not yield substantive differences.

Finally, the pictographic scenarios contained limited explicit information, and study participants’ responses likely would have been conditioned on their interpretation of the pictographs. For example, images did not contain detail on child age. It is possible that caregivers may have selected different discipline strategies if they perceived the age of the depicted child to be younger vs. older. It is also possible that some study participants could have made assumptions about the age of the child depicted, and this could have informed their responses differentially compared with other study participants. Consistent with this possibility, research assistants did report that some participants tried to ask about the age of the children in the images before providing their response, and that other study participants provided long justifications (verbally) for their selections prior to making a final answer. However, we note that, in order for any putative perceptual differences to bias our estimates toward or away from the null, study participants’ perceptions of the depicted children’s ages would need to be *systematically* different, on average, according to the variables of interest.

For example, study participants shown the scenes from the home setting would need to systematically perceive the depicted children’s ages to be older or younger compared with study participants shown the scenes from the market setting. Alternatively, women participants would need to systematically perceive the depicted children’s ages to be older or younger compared with men participants. Based on our knowledge of the participants and the study setting, we can think of no factors that can provide a reasonable explanation for why this might be the case.

### Conclusions

In this study, we found a high prevalence of physically harsh discipline preferences among caregivers in rural Uganda, with a higher prevalence seen among women and in household settings. While we did not find associations between measures of economic security and physically harsh discipline preferences, the high prevalence may broadly reflect poverty in this setting, as well as pervasive social norms and attitudes around child discipline. Given links between physically harsh discipline and poor developmental outcomes, family interventions that provide education and alternative discipline strategies are warranted.

## Supplementary Material

Suppl Files 1-3

## Figures and Tables

**Table 1:** Descriptive statistics of participant sample, stratified by caregiver sex

	Caregiver Sex					
	Female (n = 152, 43.4%)		Male (n = 198, 56.6%)		Total (n = 350)	
	n	%	n	%	n	%
**Age (years)**						
Mean (SD)	35.4 (8.60)		43.1 (9.95)		39.7 (10.1)	
**Education**						
Completed primary school	88	57.9%	133	67.2%	221	63.1%
Did not complete primary School	64	42.1%	65	32.8%	129	36.9%
**Married**						
Yes	133	87.5%	188	95.0%	321	91.7%
No	19	12.5%	10	5.05%	29	8.29%
**Religion**						
Protestant	105	69.1%	150	75.8%	255	72.9%
Catholic	33	21.7%	33	16.7%	66	18.9%
Born Again Pentecostal	14	9.21%	12	6.06%	26	7.43%
Other	0	0%	3	1.52%	3	0.86%
**Household Food Insecurity**						
Food secure	56	36.8%	82	41.4%	138	39.4%
Mild food insecurity	15	9.87%	28	14.1%	43	12.3%
Moderate food insecurity	60	39.5%	76	38.4%	136	38.9%
Severe food insecurity	21	13.8%	12	6.06%	33	9.43%
**Household Water Insecurity**						
Water secure	96	63.2%	128	64.7%	224	64.0%
Mild water insecurity	16	10.5%	18	9.09%	34	9.71%
Moderate water insecurity	20	13.2%	25	12.6%	45	12.9%
Severe water insecurity	20	13.2%	27	13.6%	47	13.4%
**Self-Perceived Relative Wealth**						
Least poor	3	1.97%	7	3.54%	10	2.86%
Better off	16	10.5%	33	16.7%	49	14.0%
Average	78	51.3%	98	49.5%	176	50.3%
Worse off	46	30.3%	44	22.2%	90	25.7%
Poorest	9	5.92%	14	7.07%	23	6.57%
Do not know	0	0%	2	1.01%	2	0.57%

Abbreviations: SD, standard deviation

Figures do not add to 100% due to rounding

**Table 2:** Prevalence of physically harsh discipline preferences (beating or slapping), stratified by caregiver sex, setting, child sex, and child behavior

	Caregiver Sex					
Setting	Female (n = 152, 43.4%)		Male (n = 198, 56.6%)		Total (n = 350)	
**Market:**	n = 54		n = 125		n = 179	
**Girl**	n	%	n	%	n	%
Whine	17	31.5%	21	16.8%	38	21.2%
Spill	19	35.2%	29	23.2%	48	26.8%
Kick	18	33.3%	40	32.0%	58	32.4%
**Any behavior**	27	50.0%	52	41.6%	79	44.1%
**Boy**						
Whine	15	27.8%	25	20.0%	40	22.3%
Spill	20	37.0%	38	30.4%	58	32.4%
Kick	28	51.9%	38	30.4%	66	36.9%
**Any behavior**	29	53.7%	61	48.8%	90	50.3%
**House:**	n = 98		n = 73		n = 171	
**Girl**	n	%	n	%	n	%
Whine	34	34.7%	16	21.9%	50	29.2%
Spill	53	54.1%	32	43.8%	85	49.7%
Kick	61	62.2%	33	45.2%	94	55.0%
**Any behavior**	81	82.7%	43	58.9%	124	72.5%
**Boy**						
Whine	32	32.7%	20	27.4%	52	30.4%
Spill	58	59.2%	31	42.5%	89	52.0%
Kick	67	68.4%	37	50.7%	104	60.8%
**Any behavior**	82	83.7%	48	65.8%	130	76.0%
**Either Setting:**	n = 152		n = 198		n = 350	
Any behavior	117	77.0%	116	58.6%	233	66.6%

Figures do not add to 100% due to rounding

**Table 3:** Adjusted negative binomial regression models estimating associations between the cumulative number of physically harsh discipline preferences and indicators of economic security

	Cumulative Number of Physically Harsh Discipline Preferences		
	Adjusted b	95% CI	*p*-value
**Household Food Insecurity**			
Food secure	*reference*		
Mild food insecurity	0.199	−0.045 to 0.443	0.109
Moderate food insecurity	0.118	−0.097 to 0.332	0.282
Severe food insecurity	0.168	−0.008 to 0.344	0.061
**Household Water Insecurity**			
Water secure	*reference*		
Mild water insecurity	0.019	−0.284 to 0.322	0.904
Moderate water insecurity	−0.065	−0.414 to 0.285	0.717
Severe water insecurity	−0.009	−0.160 to 0.143	0.911
**Household Asset Wealth Quintile**			
Richest	*reference*		
2nd	0.077	−0.186 to 0.341	0.565
3rd	−0.017	−0.395 to 0.361	0.930
4th	−0.022	−0.299 to 0.254	0.930
Poorest	0.206	−0.007 to 0.425	0.066
**Self-Perceived Relative Wealth**			
Least poor	*reference*		
Better off	−0.340	−0.671 to −0.010	0.044
Average	−0.080	−0.284 to 0.123	0.439
Worse off	−0.334	−0.631 to −0.038	0.027
Poorest	−0.224	−0.662 to 0.214	0.316

Each of the four models is adjusted for age, caregiver sex, primary school completion, marital status, and setting

## Data Availability

Data supporting the findings of this study are available from the corresponding author ENS upon request.
