# Supplementary material for: Caregiver preferences for physically harsh discipline of children in rural Uganda
Source: J Fam Violence. Author manuscript; Available in PMC 2025 Jul 1. (PMC11218336; doi:10.1007/s10896-023-00536-4)
Supplement: Suppl Files 1-3 [file NIHMS1918808-supplement-Suppl_Files_1-3.docx]

**Supplementary File 1.** Inverse probability weighted estimates of the prevalence of physically harsh discipline preferences (beating or slapping), stratified by caregiver sex, setting, child sex, and child behavior.

|  | **Caregiver Sex** | |
| --- | --- | --- |
| **Setting** | **Female** | **Male** |
| **Market:** | | |
| **Girl** |  |  |
| Whine | 37.0% | 17.5% |
| Spill | 34.9% | 23.1% |
| Kick | 42.1% | 30.8% |
| **Any behavior** | 55.3% | 40.0% |
| **Boy** |  |  |
| Whine | 31.8% | 17.8% |
| Spill | 36.8% | 29.4% |
| Kick | 54.4% | 28.7% |
| **Any behavior** | 53.2% | 45.8% |
| **House:** | | |
| **Girl** |  |  |
| Whine | 32.4% | 22.3% |
| Spill | 53.9% | 39.8% |
| Kick | 58.9% | 42.8% |
| **Any behavior** | 79.3% | 56.9% |
| **Boy** |  |  |
| Whine | 35.9% | 26.0% |
| Spill | 59.8% | 39.7% |
| Kick | 64.6% | 49.9% |
| **Any behavior** | 82.7% | 65.5% |
| **Either Setting:** | | |
| Any behavior | 70.1% | 43.8% |

**Supplementary File 2.** Adjusted negative binomial regression models with inverse probability weights estimating associations between the cumulative number of physically harsh discipline preferences and indicators of economic security.

|  | **Cumulative Number of Physically Harsh Discipline Preferences** | | |
| --- | --- | --- | --- |
|  | **Adjusted b** | **95% CI** | ***p*-value** |
| **Household Food Insecurity** |  |  |  |
| Food secure | *reference* |  |  |
| Mild food insecurity | 0.121 | -0.250 to 0.491 | 0.524 |
| Moderate food insecurity | 0.129 | -0.047 to 0.305 | 0.150 |
| Severe food insecurity | 0.311 | 0.087 to 0.536 | 0.007 |
| **Household Water Insecurity** |  |  |  |
| Water secure | *reference* |  |  |
| Mild water insecurity | 0.143 | -0.196 to 0.481 | 0.409 |
| Moderate water insecurity | 0.011 | -0.400 to 0.422 | 0.957 |
| Severe water insecurity | -0.015 | -0.214 to 0.185 | 0.886 |
| **Household Asset Wealth Quintile** |  |  |  |
| Richest | *reference* |  |  |
| 2nd | 0.010 | -0.333 to 0.353 | 0.953 |
| 3rd | 0.138 | -0.338 to 0.614 | 0.569 |
| 4th | -0.039 | -0.419 to 0.341 | 0.841 |
| Poorest | 0.219 | -0.111 to 0.548 | 0.193 |
| **Self-Perceived Relative Wealth** |  |  |  |
| Least poor | *reference* |  |  |
| Better off | -0.306 | -0.674 to -0.062 | 0.103 |
| Average | 0.002 | -0.251 to 0.255 | 0.987 |
| Worse off | -0.165 | -0.506 to 0.176 | 0.344 |
| Poorest | -0.272 | -0.720 to 0.175 | 0.233 |
| Each of the four models is adjusted for age, caregiver sex, primary school completion, marital status, and setting | | | |

**Supplementary File 3.** Adjusted negative binomial regression models estimating associations between the cumulative number of physically harsh discipline preferences and indicators of economic security, stratified by child sex.

|  | **Cumulative Number of Physically Harsh Discipline Preferences** | | | | | |
| --- | --- | --- | --- | --- | --- | --- |
|  | **Boys** | | | **Girls** | | |
|  | **Adjusted b** | **95% CI** | ***p*-value** | **Adjusted b** | **95% CI** | ***p*-value** |
| **Household Food Insecurity** |  |  |  |  |  |  |
| Food secure | *reference* |  |  | *reference* |  |  |
| Mild food insecurity | 0.222 | 0.007 to 0.437 | 0.043 | 0.090 | -0.222 to 0.402 | 0.571 |
| Moderate food insecurity | 0.116 | -0.075 to 0.308 | 0.234 | 0.119 | -0.129 to 0.367 | 0.348 |
| Severe food insecurity | 0.195 | -0.036 to 0.425 | 0.098 | 0.108 | -0.064 to 0.280 | 0.219 |
| **Household Water Insecurity** |  |  |  |  |  |  |
| Water secure | *reference* |  |  | *reference* |  |  |
| Mild water insecurity | 0.153 | -0.103 to 0.409 | 0.241 | -0.103 | -0.445 to 0.239 | 0.554 |
| Moderate water insecurity | -0.021 | -0.394 to 0.353 | 0.913 | -0.110 | -0.451 to 0.231 | 0.526 |
| Severe water insecurity | 0.033 | -0.111 to 0.177 | 0.652 | -0.108 | -0.304 to 0.087 | 0.277 |
| **Household Asset Wealth Quintile** |  |  |  |  |  |  |
| Richest | *reference* |  |  | *reference* |  |  |
| 2nd | 0.089 | -0.155 to 0.334 | 0.474 | 0.087 | -0.198 to 0.372 | 0.551 |
| 3rd | -0.015 | -0.382 to 0.352 | 0.936 | -0.058 | -0.444 to 0.327 | 0.767 |
| 4th | 0.001 | -0.231 to 0.234 | 0.990 | -0.012 | -0.322 to 0.299 | 0.941 |
| Poorest | 0.252 | 0.074 to 0.430 | 0.005 | 0.157 | -0.078 to 0.391 | 0.191 |
| **Self-Perceived Relative Wealth** |  |  |  |  |  |  |
| Least poor | *reference* |  |  | *reference* |  |  |
| Better off | -0.310 | -0.571 to -0.050 | 0.019 | -0.411 | -0.833 to 0.010 | 0.056 |
| Average | -0.106 | -0.363 to 0.151 | 0.420 | -0.071 | -0.492 to 0.350 | 0.741 |
| Worse off | -0.286 | -0.666 to 0.094 | 0.140 | -0.378 | -0.756 to 0.001 | 0.050 |
| Poorest | -0.213 | -0.595 to 0.169 | 0.274 | -0.195 | -0.849 to 0.459 | 0.558 |
| Each of the four models is adjusted for age, caregiver sex, primary school completion, marital status, and setting | | | | | | |
